# Unexpected Interaction with Dispersed Crude Oil Droplets Drives Severe Toxicity in Atlantic Haddock Embryos

**DOI:** 10.1371/journal.pone.0124376

**Published:** 2015-04-29

**Authors:** Elin Sørhus, Rolf B. Edvardsen, Ørjan Karlsen, Trond Nordtug, Terje van der Meeren, Anders Thorsen, Christopher Harman, Sissel Jentoft, Sonnich Meier

**Affiliations:** 1 Institute of Marine Research, Nordnes, Bergen, Norway; 2 Centre for Ecological and Evolutionary Synthesis (CEES), University of Oslo, Blindern, Oslo, Norway; 3 SINTEF Materials and Chemistry, Sluppen, Trondheim, Norway; 4 Norwegian Institute for Water Research (NIVA), Grimstad, Norway; 5 Institute of Marine Research (IMR), Austevoll Research Station, and Hjort Centre for Marine Ecosystem Dynamics, Storebø, Norway; University of Siena, ITALY

## Abstract

The toxicity resulting from exposure to oil droplets in marine fish embryos and larvae is still subject for debate. The most detailed studies have investigated the effects of water-dissolved components of crude oil in water accommodated fractions (WAFs) that lack bulk oil droplets. Although exposure to dissolved petroleum compounds alone is sufficient to cause the characteristic developmental toxicity of crude oil, few studies have addressed whether physical interaction with oil micro-droplets are a relevant exposure pathway for open water marine speices. Here we used controlled delivery of mechanically dispersed crude oil to expose pelagic embryos and larvae of a marine teleost, the Atlantic haddock (*Melanogrammus aeglefinus*). Haddock embryos were exposed continuously to two different concentrations of dispersed crude oil, high and low, or in pulses. By 24 hours of exposure, micro-droplets of oil were observed adhering and accumulating on the chorion, accompanied by highly elevated levels of *cyp1a*, a biomarker for exposure to aromatic hydrocarbons. Embryos from all treatment groups showed abnormalities representative of crude oil cardiotoxicity at hatch (5 days of exposure), such as pericardial and yolk sac edema. Compared to other species, the frequency and severity of toxic effects was higher than expected for the waterborne PAH concentrations (e.g., 100% of larvae had edema at the low treatment). These findings suggest an enhanced tissue uptake of PAHs and/or other petroleum compounds from attached oil droplets. These studies highlight a novel property of haddock embryos that leads to greater than expected impact from dispersed crude oil. Given the very limited number of marine species tested in similar exposures, the likelihood of other species with similar properties could be high. This unanticipated result therefore has implications for assessing the ecological impacts of oil spills and the use of methods for dispersing oil in the open sea.

## Introduction

Worldwide energy demands have resulted in increased hydrocarbon extraction activity in Polar Regions, as well as at greater ocean depths. This includes areas around the Lofoten Islands, in the Norwegian Arctic and in the Barents Sea, which are all regions considered to be especially vulnerable since they are important spawning and nursing grounds for several commercially important species of marine fish, including Atlantic haddock (*Melanogrammus aeglefinus)*, Atlantic cod (*Gadus morhua*) and herring (*Clupea harengus*) [1]. Consequently, there is great concern surrounding the possible long-term impact on the ecosystems in these areas from either accidental oil spills or from chronic exposure to operational discharges of produced water [2–5].

To be able to predict any outcome of an eventual oil spill, comprehensive risk assessment tools are needed. In recent years large efforts have been put into development and improvement of risk assessment tools by coupling different models such as, i) oil drift and fate models, ii) distribution models for zooplankton and fish embryo/larvae, iii) ecotoxicology effect models and eventually iv) ecosystem models to simulate effects of oil pollution on fish populations or multi species ecosystems [6,7]. To reach this ambitious goal however, there is a clear need for more and better empirical data in all steps of the modelling. Especially for the ecotoxicity models there are still clear limitations in the available data on relevant marine coldwater species. In particular there is a need for more data on fitness parameters (survival, development and growth) and bioaccumulation in early life stages of marine fish in order to produce reliable risk models [8]. For example fish embryo and yolk sac larvae are sensitive to low total polyaromatic hydrocarbons (TPAH) concentrations (0.1–50 μg/l), and pericardial or yolk sac edema, bone deformities, delayed development or mortality have been observed [9–16].

There is a general understanding that it is primarily the water dissolved oil compounds which provide the largest contribution to the toxicity to fish, as they are more readily bioavailable [13,17]. This may have important implications for which compounds (only the more readily water soluble PAHs or the total oil load) that are used in the risk assessment modelling of oil spills [7]. Catastrophes like the 1989 *Exxon Valdez* and 2010 *Deepwater Horizon* oil spills led to intensive studies on the effects of crude oils on developing fish. Field-collected herring and salmon larvae after the *Exxon Valdez* showed developmental defects and mortality, which was linked to PAHs, an abundant fraction of most crude oils [9,18,19]. Further research using the zebrafish model proposed that the crude oil caused disruption of embryonic cardiac function and morphogenesis. This cardiotoxicity was specifically associated to three-ringed PAHs [20,21], and it is now established that crude oil from various geological sources disrupts heart development [22,23] in diverse fish species [24,25]. Recent studies imply that compounds in the oil have the ability to block potassium and calcium ion channels, thereby disrupting signaling essential for excitation-contraction coupling in heart muscle cells [25].

When oil is released into the sea, wave energy and/or use of chemical dispersant during clean up may generate oil-in-water dispersions with micron-sized oil droplets that persist in the water column [13]. A study aiming to investigate the direct effect of different oil fractions (crude or weathered oil, and shaken or sonicated water accommodated fractions (WAFs)), during early life stages of the medaka (*Oryzias latipes*) [26], suggests that direct contact with oil leading to an accumulation on the chorion (eggshell) resulted in an enhanced toxicity not observed in exposures to WAF [26]. Additionally, while the toxicity of exposures using the WAF of weathered oils have predominantly been explained by the PAH concentrations, significant contributions from both unknown and less hydrophobic compounds have also been reported. Thus, in order to adequately ascertain the toxicity posed by oils spills, exposure studies should be designed to also include the potential for enhanced uptake from oil droplets [27–29].

The Atlantic haddock is a teleost belonging to the Gadidae family, and is a commercially important marine fish distributed on both sides of the North Atlantic [1]. Spawning occurs at depths of 30–500 m or even deeper and the fertilized eggs rise to the surface where they are subject to transport by water currents [1,30]. The haddock eggs have high buoyancy, thus the majority of eggs are found in the upper 20 m in the water column [31].

The major undertaking of this study was to obtain more realistic toxicity data on early life stages of Atlantic haddock exposed to an oil dispersion containing a combination of dissolved WAF and oil micro-droplets. The exposure was done with an oil-in-water dispersion in two concentrations, low (130 μg oil/L) and high (1200 μg oil/L), which are environmentally relevant after a large oil spill [7]. In an actual oil spill, it is likely for fish eggs/larvae to experience intermittent or transient exposure as a result of vertical transport in and out of high oil concentration areas. This study therefore included a pulse exposure: 1200 μg oil/L for 2.4 hours in a 24 hour period (the low and pulse treatments groups are exposed to the same total amount of oil over time). Assessment parameters were accomplished by fitness measurements (survival and growth) and recording phenotypic and histological abnormalities as well as examining the expression of genes involved in the detoxification pathway, especially *cyp1a*.

## Materials and Methods

### Animal collection, maintenance and exposure set up

A wild broodstock population of 61 maturing individuals was collected February-March 2013 at spawning grounds in the Austevoll area, on the west coast of Norway, and kept in two 7000 L tanks at the Institute of Marine Research (IMR), Austevoll Research station. The haddock spawns voluntarily in captivity, and fertilized eggs could therefore be collected from the tanks, transferred to indoor egg incubators, and maintained at 7.0°C until ten days post fertilization (dpf). At 10 dpf, ≈6000 eggs were transferred into each of twelve 50 L circular exposure tanks of green PE plastic (S1 Fig). The flow through the tanks was 32 L/hr, the water temperature 8.0°C, and light regime was 12D:12L. Light for triplicate tanks was provided by the broad spectrum 2x36W Osram Biolux 965 (Munich, Germany, www.osram.com) dimmable fluorescent light tubes with 30 min. smooth transitions between light and dark. From four days post hatching (dph), natural zooplankton, mainly copepod nauplii of *Acartia longiremis*, was harvested from the marine pond system “Svartatjern” [32] and introduced as feed to the larvae.

The tanks were further supplemented with marine microalgae concentrate (Instant Algae, Nanno 3600, Reed Mariculture Inc., CA, USA) until termination of the experiment [33,34]. The embryos started to hatch at 13 dpf and 50% hatch was observed at 14 dpf (= 0 dph). The exposure was stopped at 14 dph and all surviving larvae were counted and transferred into new 50 L tanks with clean seawater for further monitoring.

### Oil exposure regime

The oil used was a weathered blend crude oil from the Heidrun oil field of the Norwegian Sea. The blend oil comes from 4 different formations that contain different oil types, both light paraffinic oils (0.83 g/ml) and heavy biodegradated oil (0.93 g/ml) and is exported as a heavy crude oil (0.89 g/ml). The Heidrun blend oil is thought to be representative for the oil types that may be found in the Lofoten area. The oil is artificially weathered by distillation to account for the fast evaporation that normally occurs after an oil spill at sea. This procedure [35] is a simple one-stage distillation to vapour temperatures of 250°C leaving a residue that corresponds to 2–7 days on the sea surface at about 10°C ambient temperature, in this case causing an evaporative loss of 24% of the lighter compounds from the fresh crude oil, and it changes the oil density from 0.89 g/mL to 0.92 g/mL (SINTEF, 2004) [36].

The principle of the exposure system and the oil droplet generation is given in detail by Nordtug et al., (2011) [37]. The oil was pumped into the dispersion system using a HPLC pump (Pharmacia, LKB2150) with a flow of 0.01 mL/min together with a flow of seawater of 180 mL/min. This system generates an oil dispersion with oil droplets in the low μm range with a nominal oil load of 46 mg/mL (stock solution). The exposure dose to the tanks was regulated by a parallel pipeline system with one line of flowing clean seawater and the other line containing a flow of the stock solution. The 2 pipelines were connected by a 3-way magnetic valve allowing water to be collected from both lines. Different dilutions were made by controlling the relative sampling time from the oil stock solution and clean water, respectively, by a computer-controlled relay (MiniBee card and BeeStep software). The experimental setup consisted of three treatments and a control, each with three replicates: 1) Low: 130 μg oil/L nominal. 2) High: 1200 μg oil/L nominal. 3) Pulse: 1200 μg oil/L nominal for 2.4 hours in a 24 hour period. 4) Control: No oil. The concentration of oil in the pulse tanks decreased to approximately zero before the next pulse (S2 Fig). The oil dispersion doses were given by opening the magnetic valve for 27 seconds every minute in the high treatment group and 3 seconds in the low treatment group. The pulse treatment group received 27 seconds of oil stock solution every minute for 2.4 hours in each 24 hour period (S2 Fig). Oil dispersions were delivered to each of the replicate exposure tanks at a flow rate of 30 mL/min and mixed into the main water supply of 500 mL/min (S1 Fig). The oil exposure lasted for 18 days (S2 Fig). To avoid oil film on the water surface in the tanks, a sharp cut oblique drains covered with a plankton mesh was mounted in all tanks, including the control tanks.

Embryos and larvae were collected at eleven different time points from 11 dpf to 17 dph in addition to an initial sample at 10 dpf. Two of the samples were collected after the exposure period (S2 Fig). All animals collected for RNA extraction were photographed in a microscope before they were snap frozen in liquid nitrogen and stored at -80°C. Two pools of embryos were collected at the first six time points for total RNA extraction. For the subsequent time points, 12 individuals were sampled for total RNA extraction.

### Analytical chemistry

Water samples from each tank (1 L) were taken before the exposure started and at the end of exposure. The water sampled were extracted with dichloromethane and prepared for analysis of total hydrocarbons (THC) and PAHs. Details of these analytical methods are given in S1 Text.

Oil droplets were diameter measured from a picture with a resolution of 1.38 pixels/μm obtained by a stereo microscope (Olympus SZX-10) at 6.3 X magnification and a 21 MPixel camera (Canon EOS 5D Mark II).

### Sampling and fitness measurements

The hatching success was estimated at 2 dph. The tanks were gently stirred to obtain an even distribution of larvae, and subsamples from top to bottom were taken with a cylinder (volume 200 mL) whereafter number of larvae was calculated. Hatching were then estimated from number of eggs stocked, subtracted the embryos sampled (≈400) during the egg phase. Survival post exogenous feeding (14 dph) was measured by calculating all surviving larvae when transferred into new 50 L tanks with clean sea water for further monitoring. The survival (%) was calculated according to the number of larvae in each tank at 2 dph.

### Analysis of phenotypic data

The length of larvae from 1 dph to 17 dph was measured using ImageJ (ImageJ v2013_2, National Institutes of Health, Bethesda, Maryland, USA). In addition, the presence of 6 deformity parameters; craniofacial deformities, jaw deformities, pericardial and yolk sac edema, spinal curvature and lack of pigmentation in a large set of larvae from each treatment and control were given a grade from 0–3 (where 0 indicates no deformity, 1 some, 2 significant and 3 severe deformity). In some animals or sampling points, some of the parameters were not visible and therefore denoted not applicable (NA) and taken out of the calculation. The proportion of deformities was calculated as follows: (Sum of observations for each grade / ((Total number of larvae*6 parameters)—Sum of NA)) *100%.

### Total RNA and cDNA preparation

Total RNA was isolated from frozen pools of animals and individual larvae (except at sampling point at 7 dph and 8 dph) using Trizol reagent (Invitrogen, Carlsbad, California, USA), according to procedures provided by the manufacturer which included a DNase treatment step using a TURBO DNA-*free* kit (Life Technologies Corporation). Total RNA from single larvae from 7 dph and 8 dph were extracted using RNeasy micro kit (QIAGEN Sample and Assay Technologies) according to procedures provided by the manufacturer. The amount of RNA was quantified using a Nanodrop spectrophotometer (NanoDrop Technologies, Wilmington, DE, USA), and quality checked using a 2100 Bioanalyzer (Agilent Technologies, Santa Clara, CA). cDNA was subsequently generated using SuperScript VILO cDNA Synthesis Kit (Life Technologies Corporation), according to the manufacturer’s instructions. The cDNA was normalized to obtain a concentration of 50 ng/μL.

### Real time qPCR

Specific primers and probes for real-time, quantitative PCR analysis of Atlantic haddock, *cyp1a*, *ahr2 and gstp1* mRNAs, as well as for the reference gene *ef1α* were designed with Primer express software (Applied Biosystems, Carlsbad, California, USA), according to the manufacturer’s guidelines. Primer and probe sequences are given in S1 Table. TaqMan PCR assays were performed in duplicate, using 384-well optical plates on an ABI Prism Fast 7900HT Sequence Detection System (Applied Biosystems, Carlsbad, CA, USA) with settings as follows: 50°C for 2 min, 95°C for 20 s, followed by a 40 cycles of 95°C for 1 s and 60°C for 20 s. For each 10 μl PCR reaction, a 2 μl cDNA 1:40 dilution was mixed with 200 nM fluorogenic probe, 900 nM sense primer, 900 nM antisense primer in 1xTaqMan Fast Advanced Master Mix (Applied Biosystems, Carlsbad, California, USA). Gene expression data was calculated relative to the start (10 dpf) sample using the ΔΔCt method as described in detail in Bogerd et. al 2001 [38].

### Histology

Five larvae from each replicate were pooled and fixed in 4% PBS buffered paraformaldehyde for 24 hrs at 4.0°C, processed using Histokinette 2000 (Reichert-Jung), and embedded in paraffin wax within three days to preserve the RNA and tissue morphology. Sample preparation was always performed under RNase free conditions. Serial sectioning (3 μm) of larvae was performed for morphological analysis or *in situ* hybridization using a Leica RM 225 microtome (Leica Microsystems). Histological sections were dewaxed and stained with haematoxylin–erythrosin–safran (HES).

For *in situ* hybridization (ISH), the Atlantic haddock cyp1a cDNA was amplified using PCR containing Sp6 and T7 primers in the forward and reverse primers, respectively (Cyp1a_FW_Sp6: 5’-ATTTAGGTGACACTATAGCATCTTCCAGATCCAGATCG -3’ Cyp1a_RV_T7: 5’-TAATACGACTCACTATAGGGCATGAACCTCTTCATGGTGG -3’). The PCR product of 501 bp was used as a template for synthesizing the sense and antisense cRNA probes by the Sp6 and T7 RNA polymerase, respectively. The digoxigenin (DIG) labelling was performed using the DIG-AP RNA labelling kit (Roche Molecular Biochemicals) following the manufacturers protocol.

The *in situ* hybridization was carried out as described by Weltzien et al. (2003) [39] with some modifications [40]. Hybridization was always carried out with sense and antisense probes on adjacent sections, and under RNase free conditions.

### Statistics

Statistical analysis was performed with GraphPad Prism, version 6 (GraphPad Software Inc., 1996, La Jolla, California, USA). Significant differences in gene expression for the time points 11 dpf—3 dph, fitness observations and analytical chemistry between control and exposure groups, were tested with a one-way ANOVA using the Dunnet’s multiple comparison test after checking for normality and variance homogeneity. Statistical analysis of differential *cyp1a* expression at time point 7 dph—17 dph and length measurements of 1 dph—17 dph were performed with a non-parametric Kruskal Wallis test using Dunn’s multiple comparisons, due to most time points not having a Gaussian distribution. A chi-square test was used to analyze for significant differences in the distribution of deformities. The level of significance was set at p<0.05 unless otherwise stated.

### Ethics Statement

All animal experiments within the study were approved by NARA, the governmental Norwegian Animal Research Authority (http://www.fdu.no/fdu/, reference number 2012/275334-2). All embryos sampled were frozen in liquid nitrogen. All larvae were euthanized using 500 mg/L MS-222 (Tricaine methanesulfonate, TS 222, Sigma-Aldrich) when sampling and at termination of the experiment to achieve immediate death. No humane endpoints were used during the experiment because the potential endpoint criteria due to their small size had to be evaluated under a microscope when they were euthanized and sampled. The animals were monitored every day, and any dead larvae were removed. The Austevoll Aquaculture Research station has the following permission for catch and maintenance of Atlantic haddock: H-AV 77, H-AV 78 and H-AV 79. These are permits given by the Norwegian Directorate of Fisheries. Furthermore, the Austevoll Aquaculture Research station has a permit to run as a Research Animal facility using fish (all developmental stages), with code 93 from the national IACUC; NARA.

## Results

### Analytical chemistry

The weathered Heidrun Blend oil used in the current study was a mixture of both light paraffinic oil and more heavily degradable oils. The PAH constituted 2% of the total oil by weight (19.6 g/kg), and the PAH profile was dominated by the C0–C3 naphthalenes (71%), followed by the tricyclic PAHs which contributed 28% of the total PAHs (S3 Fig).

**Fig 1:**
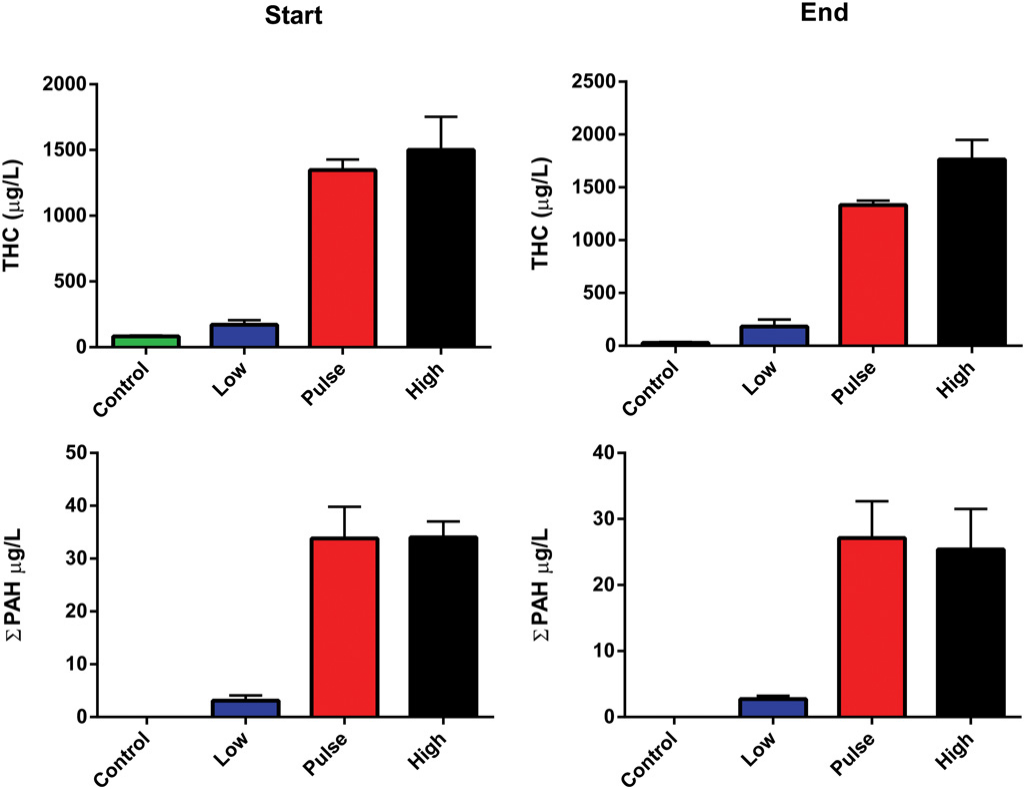
Water analysis of total hydrocarbon (THC) and total polyaromatic hydrocarbons. (sum of 19 PAHs and C1–C3 alkylated isomers of naphthalene, phenanthrene, dibenzothiophene and chrysene) at the exposure start and the exposure end. A detailed list of the PAHs included in the analysis are given in the supplementary information. The results are presented as average numbers (± SD) from one analysis from each of the 3 tanks from all treatments. The concentration in the pulse dose treatment was measured at the maximum of the pulse dose after 2.4 hours oil exposure.

Haddock embryos and larvae were exposed to a mixture containing both oil micro-droplets (diameter 18±3 μm) and WAF. The total oil concentrations in the tanks were measured at the start of the experiment to; 170 μg THC/L and 3 μg TPAH/L in the low dose group and 1500 μg THC/L and 34 μg TPAH/L in the high dose. The pulse group had similar concentrations to the high dose when measured at the end of the 2.4 hr pulse. Average concentrations recorded at the end of the 18 days exposure were within 75% of the initial concentration (26 μg TPAH/L in the high and pulse groups and 2.7 μg TPAH/L in the low dose, Fig 1). The PAH profile in the exposure tanks had slightly higher levels of naphthalene and C1–C4 alkylated naphthalenes (75% of TPAH) compared with pure oil (71%, S3 Fig).

### Phenotypic and fitness observations

**Fig 2:**
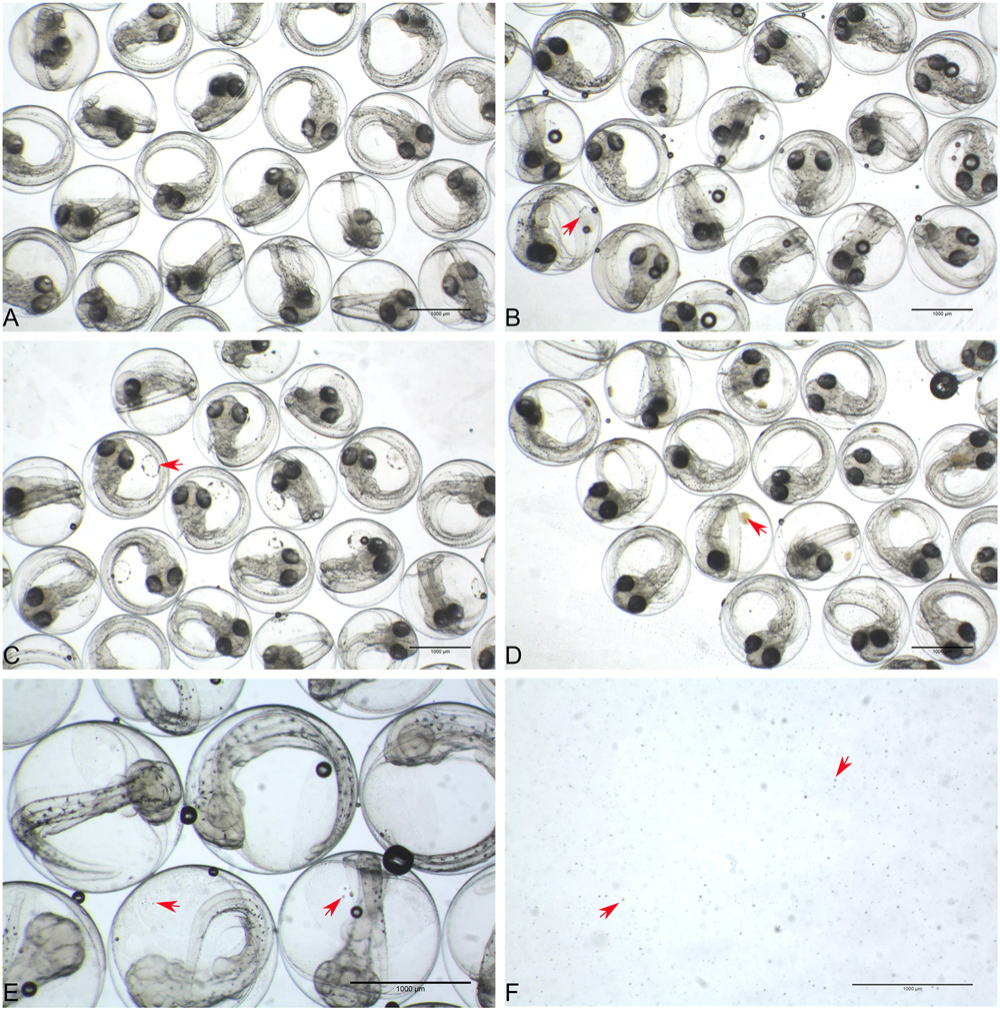
Attraction of micro-droplets to chorion. Embryos after 4 days of exposure: (A) Control. (B) Low dose. (C) Pulse dose. (D) High dose. Arrows indicate examples where attraction of micro-droplets appears to be more pronounced. (E) Oil droplets seen on the chorion. (F) Oil droplets in oil dispersion stock solution. The stock solution was diluted 39 and 265 times to obtain high and low dose, respectively.

The first observation was a general higher buoyancy of the oil exposed haddock embryos and attraction of oil micro-droplets to the chorion (Fig 2). This was seen as early as 24 hours of exposure (S4 Fig), but the attraction was more evident after 4 days due to a localized accumulation on the chorion. Oil micro-droplets accumulated in a ring formation in the pulse and low dose groups, but appeared to coalesce into a larger mass on embryos in the high dose group. The micro-droplets seen on the chorion were similar size as the oil micro-droplets in oil dispersion (Fig 2).

**Fig 3:**
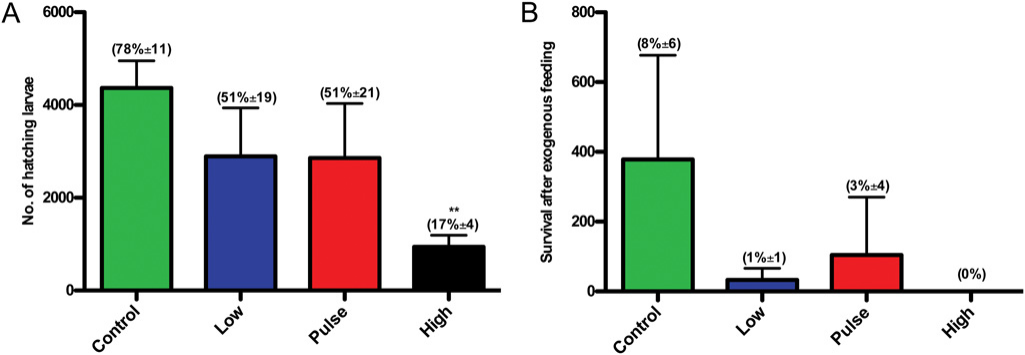
Fitness observations. (A) Average number of hatching larvae after 7 days exposure at embryo stages (start incubation of 6000 embryo in each tank) and (B) Number of surviving larvae after 10 days of exogenous feeding (14 dph, total 18 days of exposure). The numbers in brackets indicate A. average hatching success in percent (±SD) and B. average larvae survival in percent after exogenous feeding (±SD). Asterisks indicate statistical significance difference to the control fish, p<0.01 = **.

The hatching success in the high dose groups was very poor (17%±4), and the majority of the larvae were severely deformed which resulted in termination of the high dose group after 8 dph. Lower hatching success and reduced survival after exogenous feeding in the low and pulse groups were observed, however, this was not significant because of the low number of replicates (n = 3) (Fig 3).

**Fig 4:**
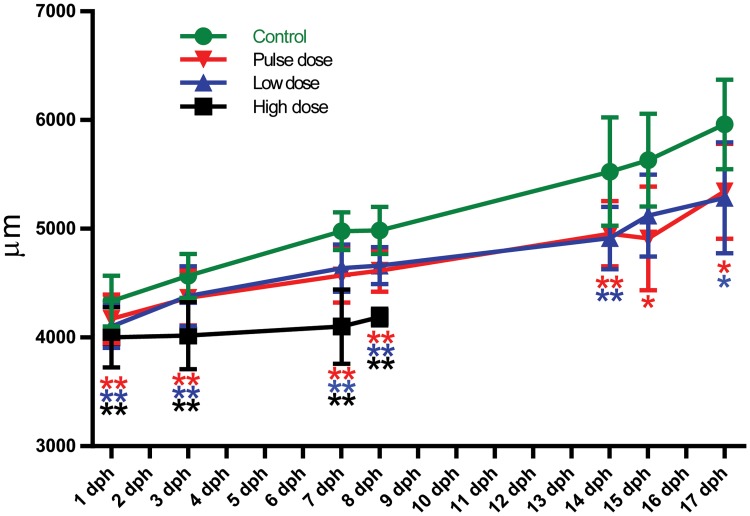
Length curve of larvae from hatching until end of experiment. Points shown as average (±SD). Asterisks indicate statistical significance difference to the control fish, p<0.05 = *, p<0.01 = **.

The newly hatched larvae in the exposed groups were significantly shorter than the larvae from the control group (Fig 4). This reduced length compared to control increased throughout the period of exogenous feeding. For the high dose larvae there was no growth during the first eight days as the larvae (plankton were given at 4 dph), did not succeed with establishment of exogenous feeding.

**Fig 5:**
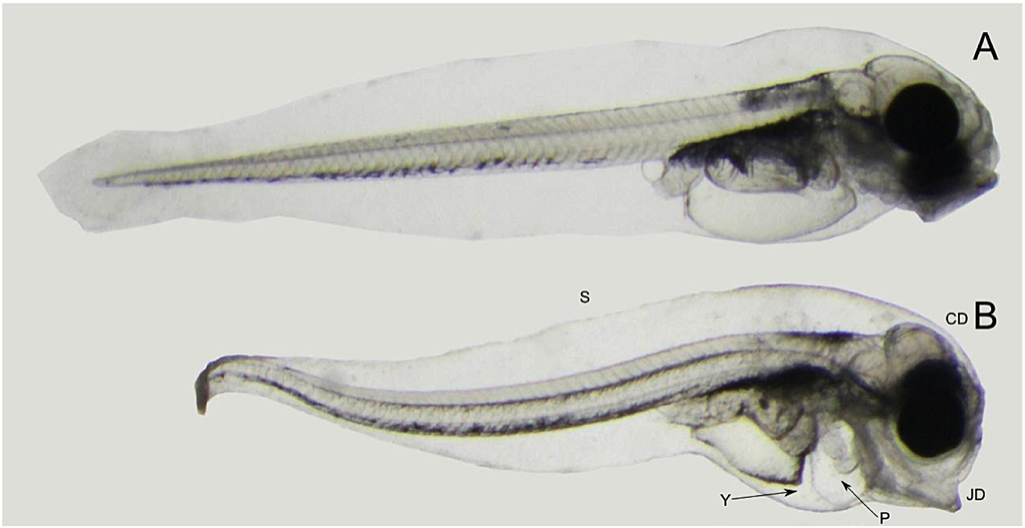
Abnormalities resulting from embryonic oil exposure. One day post hatch, 7 days of exposure. (A) Control. (B) and (C) Low dose group. Abnormalities are indicated: Pericardial edema (P), yolk sac edema (Y), spinal curvature (S), craniofacial deformities (CD), jaw deformities (JD).

**Fig 6:**
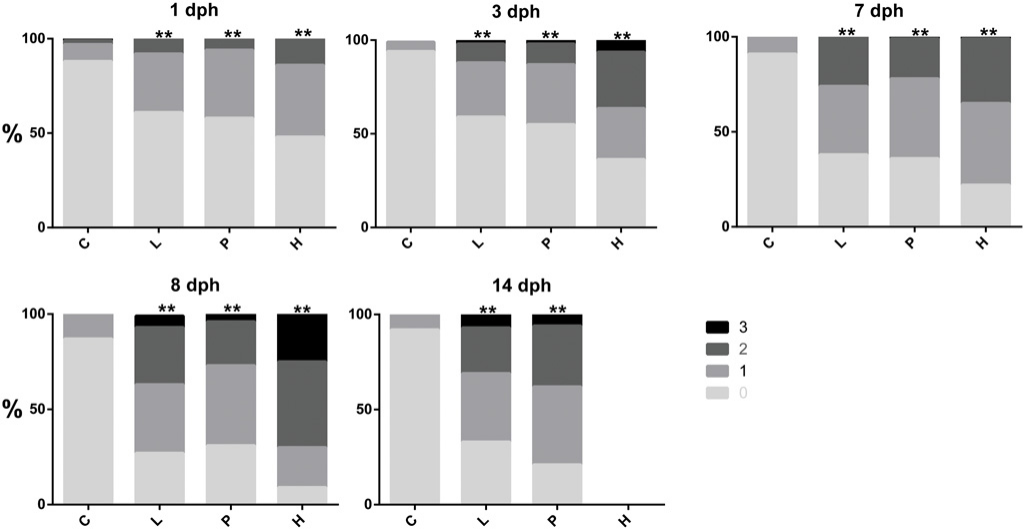
Distribution of deformities after hatch until end of exposure. 0: No deformity, 1: Some deformity, 2: Significant deformity and 3: Severe deformity. The figure shows the combined score for all six deformity parameters. Statistical difference between the treatment groups and control were analysed using chi-square test and statistical difference is indicated by asterisk, p<0.01 = ** (Detailed distribution of the single deformities are given in S2 Table).

After 7 days of exposure newly hatched haddock larvae showed morphological defects such as pericardial and yolk sac edema, malformation involving the jaws and other craniofacial structures, and dorsally curved trunks and tails (Fig 5), and increased mortality was observed in all exposed groups. The percentage of pericardial edema observed at 8 dph were 100, 81 and 100% in low dose, pulse dose and high dose, respectively, opposed to 12% in control (S2 Table). Similarly, occurrence of yolk sac edema was 83, 81, and 100% in low, pulse, and high treatment groups, compared to 12% in controls. Deformities were more frequent in the high dose group and increased with prolonged exposure time. The high dose tanks were terminated after sampling at 8 dph, and are therefore not represented at 14 dph sampling. Distribution of the presence and severity of deformities in the treatment groups and control group over time is illustrated in Fig 6 and S2 Table.

### Expression of *cyp1a*

**Fig 7:**
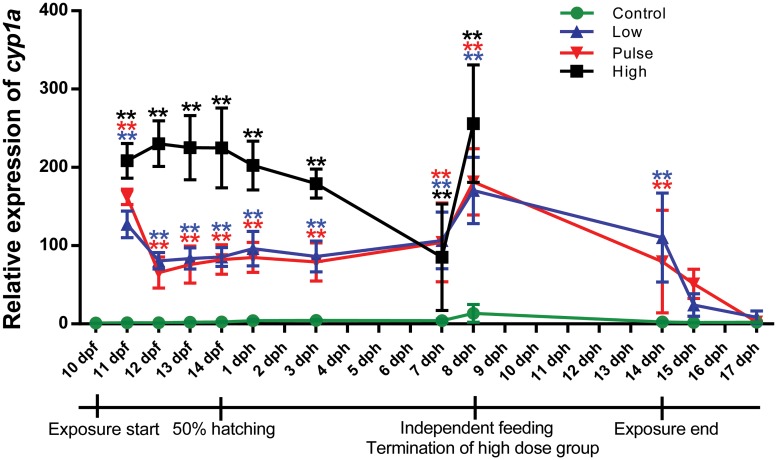
The relative expression of *cyp1a* in control, low dose, pulse dose and high dose groups at various sampling points. The expression is relative to the 0-sample (10 dpf). Fourteen dpf was set as 50% hatching, however only embryos were analysed at this time point. Oil exposure ended at 14 dph. Points are shown as average *cyp1a* expression (±SD). There are statistical differences between all treatment groups and control at all time points, except between control and pulse dose groups at 15 dph (1 day post exposure stop), and control and both low dose and pulse dose at 17 dph. Asterisks indicate statistical significance difference to the control fish, p<0.05 = *, p<0.01 = **.

An immediate response in expression of *cyp1a* to the oil exposure was observed. Compared to the control during the embryonic phase, the high group showed 200 fold up-regulated expression, and the pulse and low dose groups showed 100 fold up-regulated expression (Fig 7). The pulse dose group was initially exposed to a high dose for 2.4 hours which is reflected in an initial response intermediate to that of the high dose and low dose group.

At 7 dph and onwards, the measurements were performed on single larva, which is reflected in the expressional variation in the time points. However, we can track the expression back to phenotypic observations in some individuals, which generally are in accordance with the expression levels in the individuals; animals with severe abnormalities having a higher expression level of *cyp1a* compared to those with fewer abnormalities (S5 Fig). The expression of *cyp1a* decreased rapidly after end of exposure, and was similar to control levels 3 days post exposure stop (Fig 7).

### Expression of *ahr2* and *GSTP1*

The response in gene expression of *ahr2* was immediate in all exposure groups, but moderate compared to the strong expression of *cyp1a* (S6A Fig). The expression of *GSTP1* was delayed and the differences between treatment groups and control were only evident starting two days after exposure (S6B Fig).

### *In situ* hybridization

**Fig 8:**
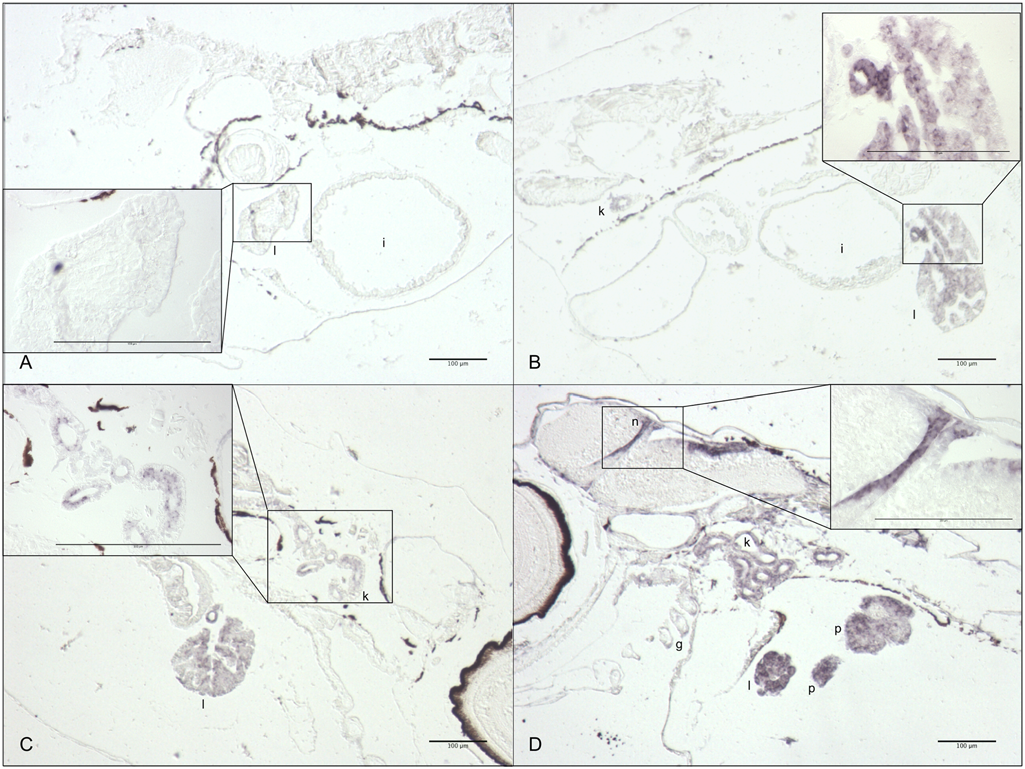
*In situ* hybridization of larvae for *cyp1a* at 8 dph, 12 days of exposure. Only antisense hybridizations are shown. **A**: Control, **B**: Low dose, **C**: Pulse dose **D**: High dose. Organs indicated: gills (g), liver (l), intestine (i), vascular endothelial cells (n), kidney (k) and pancreas (p).

**Fig 9:**
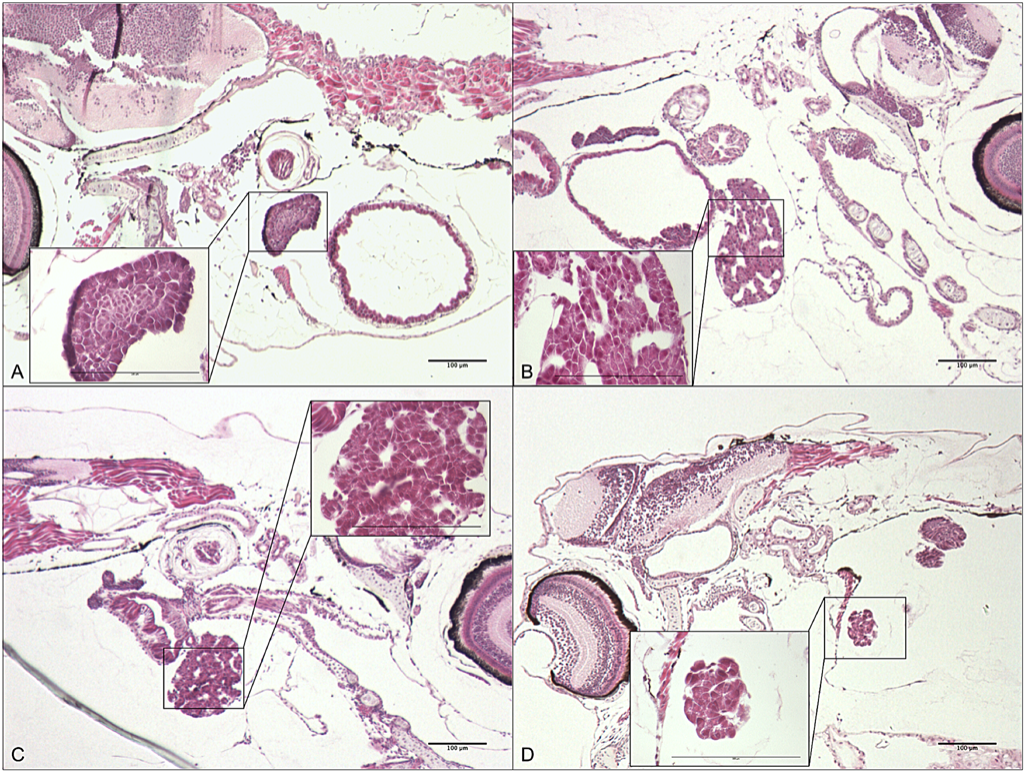
HES staining of larvae at 8 dph, 12 days of exposure. A-D show an overview of larvae from the various treatment groups and control, with the liver boxed in and magnified. **A**: Control. **B**: Low dose. **C**: Pulse dose. **D**: High dose. The extended white areas in the livers of the exposed animals indicate lesions.

*In situ* hybridization was performed to verify the expression and localization of *cyp1a* in 8 dph larvae. Even though ISH was not used quantitatively, differences in expression levels in the various treatment groups were clear (Fig 8). No *cyp1a* expression was observed in the control group. Low and pulse dose groups showed moderate expression in the liver, kidney (Fig 8B and 8C) and intestine (Fig 8B), while the high dose group showed high expression in the liver, pancreas, gills and in a layer of nucleated cells at the midbrain-hindbrain junction and adjacent to the hindbrain ventricle (n) (Fig 8D). The morphology of the liver was assessed after HES staining in sections adjacent to those used for ISH. Lesions in the liver tissue were observed in all the treatment groups, but not in the control (Fig 9A, 9B, 9C and 9D respectively).

## Discussion

The major finding from this study is amplified embryotoxicity observed in Atlantic haddock compared to similar species when exposed to dispersed crude oil during early developmental stages. In this study, haddock embryos exposed to low and high TPAH concentrations of 3 μg/L and 34 μg/L showed a suite of abnormalities typical of crude oil cardiotoxicity in other fish species: yolk sac and pericardial edema, craniofacial malformation, and curling of the body axis. Importantly, at 8 dph presence of the deformity parameters were similar in animals exposed to either 3 or 34 μg/L TPAH, although the high group had generally a higher severity grade (S2 Table and Fig 6). Pericardial edema was observed in 100% of the analysed animals exposed to 3 μg/L TPAH, indicating a severe effect even at the low concentration. Similarly, the high levels of *cyp1a* mRNA induction in the 3 μg/l exposure (~ 100-fold) relative to the 34 μg/L exposure group (~ 200-fold) is consistent with an already high tissue PAH dose in the 3 μg/L (low) treatment group in the embryo phase. Our data indicate that haddock embryos showed a more severe toxic response to this range of TPAH concentrations than anticipated from other species exposed to crude oil WAFs containing dispersed micro-droplets. For example, Pacific herring embryos exposed to a high-energy mechanical dispersion of crude oil containing droplets showed 35% yolk sac edema at 17.3 μg/L TPAH [41]. Embryos of amberjack and yellowfin tuna, both species with a buoyant pelagic egg like haddock, showed an occurrence of pericardial edema at 57 ± 10% at 13.8 μg/ and 75± 12% at 3.4 μg/L TPAH, respectively [16]. Although the response of yellowfin tuna approaches that of haddock, tuna eggs would be expected to accumulate dissolved PAHs more rapidly both because they are much smaller (~ 1 mm compared to 1.5 mm for haddock) with a higher surface to volume ratio, and because they incubate at a much higher temperature (27°C vs. 8°C). Based on egg size, haddock might be anticipated to show effects in the same range as herring or amberjack. Although we did not measure tissue PAH concentrations, none of these different species would be expected to have large differences in PAH toxicokinetics at the embryonic stage [42]. Thus the key difference in these cross-species comparisons is that adherance of oil droplets to the chorion was not observed in any of these prior studies.

The most likely explanation for the high frequency and severity of defects and high levels of *cyp1a* induction at such a low TPAH concentration (3μg/L) is the accumulation of oil micro-droplets on the chorion of haddock, which has been described as being generally more adherent than similar species like Atlantic cod [43,44]. The accumulation of micro-droplets most likely resulted in an amplified uptake of toxic compounds such as PAHs. This phenomenon was seen in all treatment groups already after 24 hours of exposure, and became more evident with prolonged exposure time. Furthermore after five days of exposure, increased mortality was observed in the exposed groups; i.e. 17% and 51% hatching success in high and low/pulse respectively compared to 78%, in the controls. These results are however, not in line with previous report on zebrafish (*Danio rerio*) [17], in which embryos were simultaneously exposed to WAFs with and without micro-droplets, indicated that dissolved PAHs alone were sufficient to cause the typical suite of defects associated with crude oil exposure and contact with droplets was not required. Moreover, in this case also there was no observation of oil micro-droplets accumulating on the chorion [17]. Additionally, toxicity of WAFs with and without droplets were similar for Atlantic cod larvae [13,45]: EC_50_ values for survival probability for cod larvae were found to be 56 μg TPAH/L for dispersed oil and 38 μg TPAH/L for WAF [13]. Adherence of oil droplets to the haddock chorion most likely leads to a dual exposure pathway: in addition to uptake of dissolved PAHs from the WAF, attached oil droplets probably either create a higher local dissolved PAH concentration at the eggshell, or potentially even lead to direct transfer of crude oil compounds across the eggshell. This interpretation is also consistent with a previous study on embryos of medaka (*Oryzias latipes*) [26] in which an enhanced toxicity was observed after direct contact with oil and accumulation on the chorion, relative to WAF exposure.

In fish, the cytochrome P450 1a (*cyp1a*) gene is an established biomarker for several aryl hydrocarbon receptor (AHR) agonists, like PAHs and selected xenobiotic compounds [46–48]. CYP1A bioactivates xenobiotics by oxidation, thus preparing them for further modification and detoxification by e.g. Glutathione S Transferases (GSTs) [49,50], particularly by GSTP1 and GSTM1 [51,52]. The gene expression data indicates an induction of the metabolism of oil components through the detoxification pathway by activation of *cyp1a* through AHR2. Accumulation of reactive intermediates further induces the expression of *GSTP1* [50] (hence the delayed response to oil exposure, S6 Fig), which modifies the reactive intermediates enabling them to be safely excreted [50,53,54]. The agreement between length, the degree of deformities and the high expression of *cyp1a* in all exposed groups indicates that the effect of the exposure is higher in the late embryo phase of Atlantic haddock than expected, based on similar studies of other species [55].

There was a considerable variation in *cyp1a* expression over time, both within and among treatments, and this variation has implications for understanding the role of droplets in exposure pathways. Over time, efficiency in hydrocarbon degradation may vary during development, i.e., embryos have been shown to have less efficient CYP1A activity from a given level of mRNA than larval stages [56]. Variation within treatment reflected individual differences, especially for the late stages from 7 dph and onward where single larvae were isolated. This was even more pronounced in the high dose group. There was also increased expression in all groups (including control) at the onset of independent exogenous feeding, consistent with an increased intestinal metabolism [57] and potentially indicating an additional route of exposure in the treatment groups. The high individual variation in cyp1a expression reflect variations in tissue PAH concentrations among individuals, which in turn could be due to a source of variability in the exposure. If embryos and larvae were taking up dissolved PAHs only, it might be expected that exposure would be more uniform, and variability in individual *cyp1a* levels would be lower. However, variability in oil droplet size adhered to the chorion or taken up through feeding could explain the higher level of exposure variability.

Despite the fact that oil components can in themselves cause toxicity in the organism, intermediates resulting from the phase 1 detoxification pathway may be more reactive, leading to an elevated toxicity caused by a cascade of downstream reactions such as oxidative stress [50,58–60]. We observed severe lesions in the liver, an organ strongly involved in detoxification and general metabolism, in the exposed groups (Figs 8 and 9), which could be a result of prolonged oil exposure. Similar lesions are seen in fish and might come from excessive storage of fat and glycogen. This phenomenon is commonly seen in cultured fish reared on artificial diets [61] and fish exposed to oil contaminants [62,63]. However, such lesions often appear as empty round cells, considering adipose tissue is lost during xylene treatment. In this case, the lesions seem to be extracellular, and may be the result of a disruption in the intracellular connection such as coagulative necrosis, fibrosis [64] or disturbance in the intercellular protein composition caused by oxidative stress[65]. Toxicity of oil and how it affects the organism is complex and much discussed [17,20,21,24,41,66–70]. As documented by other studies [71–75] and partly in the ISH in this study *cyp1a* seems to be expressed in tissues which act as first barrier organs (epidermis/gills/intestine), in tissues involved in metabolism (kidney/liver/gall bladder/pancreas), and in vascular endothelial cells including cardiac endothelium. Although toxic intermediates from the CYP1A activity could play a role in the presence of liver lesions in the exposed groups, recent data demonstrated crude oil WAFs directly disrupt ion currents necessary for excitation-contraction coupling in fish cardiomyocytes [25]. However, a role for metabolism of PAHs in cardiotoxicity has not been completely ruled out, in particular for metabolism by endocardial cells that are almost always a site of robust CYP1A induction in oil exposed fish embryos. It was also shown that the exposed groups had decreased growth compared to the control and that none of the high dose groups larvae were able to start exogenous feeding. Deformation, unsuccessful exogenous feeding and death through starvation are also reported in Atlantic cod larvae that were exposed for produced water (TPAH = 2 μg/L) during embryo stages [55]. Impaired growth after low dose oil exposure is found in both cod and Atlantic herring larvae [11,13].

The increased effect of dispersed oil on haddock embryos in the present study may have important ecological implications in oil contamination scenarios because accumulation of oil droplets on the chorion may lower the exposure time and concentration sufficient to cause toxicity. Moreover, our data do suggest that even a short exposure to a high concentration of dispersed oil may continue to affect the embryos even after they have been transferred into non-contaminated water by carrying along attached oil droplets as a continued source of exposure. Further studies are required to elucidate this potential for direct mass transfer of PAH from adhered oil droplets to the embryo. Besides achieving detailed knowledge of the mechanisms of such transfer of PAH, the extent to which this phenomenon is species specific is of importance for ecological assessment of oil exposure. Taken together, our results illustrate that in order to obtain environmentally realistic exposure experiments for early life stages it is of high importance to include dispersed oil and not only dissolved components of WAF It is also important to clarify if the use of chemical dispersants may further contribute to the increased toxicity of micron-sized oil droplet. Several laboratory studies have found that dispersants increased the concentration of toxic oil compounds within the water column both as micron-sized oil droplets and dissolved compounds, rather than synergistic toxicity from the combination of oil and oil dispersant [76,77]. Incorporation of dispersant surfactants into oil droplets could influence how those droplets interact with embryos of haddock, or other species as well.

In an oil spill scenario the vertical distribution of fish eggs and larvae will be crucial for the probability of being severely contaminated, and in the field it is likely for eggs and larvae to experience intermittent exposure as a result passage through areas with variable oil concentration [7]. The pulse exposure here was designed to examine if the most important factor for toxicity is the total dose (the low and pulse treatments groups are exposed to the same amount of oil over time) or the peak concentration (the pulse group was given a similar dose as the high group in 2.4 h every day). We observed no differences in response between the low and the pulse treatments, suggesting that the total exposure is more important than peak concentration. However, it must be pointed out that even short duration exposures of haddock embryos to dispersed oil can have severe consequences, because the accumulation of oil droplets prolongs the exposure. Finally, the potential for enhanced PAH accumulation even with short exposures leading to oil droplet accumulation could render haddock embryos particularly susceptible to the possible effect of phototoxicity in these pelagic embryos. As stated in several studies [78–80], UV radiation is found to markedly enhance the toxicity for some PAHs, and therefore relevant to include in future studies.

The present study has used data from the new risk assessment model, which couples an oil drift model and a fate model (SYMBIOSES) [7], for estimating a more realistic exposure scenario for early life stages of haddock (embryo and larvae) after a blow-out from four hypothetical platform localities around the Lofoten area. The models formed the basis to design the exposure regimes that were used in the laboratory exposure experiments. Nevertheless, it is evident that even the lowest exposure doses in the current study are too high to obtain No Observed Effect Concentration (NOEC) and therefore further experiments especially on the early embryonic phase using lower exposure doses are needed.

## Conclusion

This study indicates that haddock embryos are highly impacted by oil exposure when using a realistic oil exposure system that includes dispersed oil droplets in addition to the water accommodated fraction. The haddock embryo is known to have an adherent chorion, which seems to attract micro-droplets of oil, creating a direct connection between the toxic components of the dispersed crude oil and the embryo, and thereby enhancing the exposure.

Observations and data obtained in this study therefore emphasize the overall importance of also considering the toxicity of the oil droplet in the risk assessment.

## Supporting Information

S1 Fig Oil exposure set up. The oil was pumped into the dispersion system using a HPLC pump (Pharmacia, LKB2150). This system generates an oil dispersant with oil droplets in the low μm ranges. The exposure dose to the tanks was regulated by a parallel pipeline system with one line with clean sea water and one line with the dispersed oil—the 2 pipelines are connected by 3-way magnetic valve which switched between oil dispersant and clean water.
(TIFF)

S2 Fig Oil exposure regime. Oil exposure started at 10 dpf and ended at 14 dph after 18 days of exposure. The twelve sampling points are indicated by E0–E11. Low dose (green): nominal doses; 130 μg oil/L. High dose (black): nominal doses 1200 μg oil/L. Pulse dose (red): nominal doses 1200 μg oil/L for 2.4 hours in a 24 hour period. Concentration of oil in the pulse tank decreased to approx. 0 before next pulse.
(TIFF)

S3 Fig Profile of PAH (including their C1–C4 alkylated isomeres). The results are given as average numbers (± SD) from one analysis from each of the 3 tanks from all treatment (L, P and H) and a pure oil sample of the weathered Heidrun oil (+250°C). Detailed abbreviations for PAHs are listed in supplementary information.
(EPS)

S4 Fig Exposure 24 hours, 11 dpf. Micro-droplets of dispersed oil adhere to the chorion, and are observed in all exposure groups after 24 hours of exposure. Arrows indicate examples of micro-droplets adhered to the chorion of the Atlantic haddock embryo.
(TIFF)

S5 Fig The relative expression of *cyp1a* vs. phenotypic observations. A: High dose larvae with fewer deformities tend to have lower expression level of *cyp1a* (1 and 2) compared to larvae with significant to severe deformities (3 and 4). The numbers indicate fold change in *cyp1a* expression compared to control. B: The graph shows the *cyp1a* expression (black curve) and the degree of deformity (red curve) for individuals with linked deformity-, *cyp1a* expression information.
(TIFF)

S6 Fig Relative expression of *ahr2* and *GSTP1*. Pooled samples of embryo and larvae from 10 dpf- 3 dph in all treatment groups and control were screened for relative expression of *ahr2* and *GSTP1*. All data is given as average (±SD). A: *AhR2*. B: *GSTP1*. Asterisks indicate statistical significance difference to the control fish, p<0.05 = *, p<0.01 = **.
(EPS)

S1 Table Primers and probes for real time qPCR. (DOCX)

S2 Table The number of larvae with deformities (% of observed larvae). The table shows the detailed numbers of the measurement that are combined in Fig 6 in the paper. All parameters have been graded 0–3, where 0 = no deformity, 1 = some deformity, 2 = significant deformity, 3 = severe deformity or as NA (not applicable) if position of larva made deformity grading difficult or impossible. * = no mouth opening yet. ** = too small to observe pericardial edema. The number shows the % of larvae that are scored from 1–3.
(DOCX)

S1 Text Analytical chemistry methods. (DOC)
