# Supplementary figures and images for: Unexpected Interaction with Dispersed Crude Oil Droplets Drives Severe Toxicity in Atlantic Haddock Embryos
Source: PLoS One. 2015 Apr 29;10(4):e0124376. doi: 10.1371/journal.pone.0124376 (PMC4414579; doi:10.1371/journal.pone.0124376)

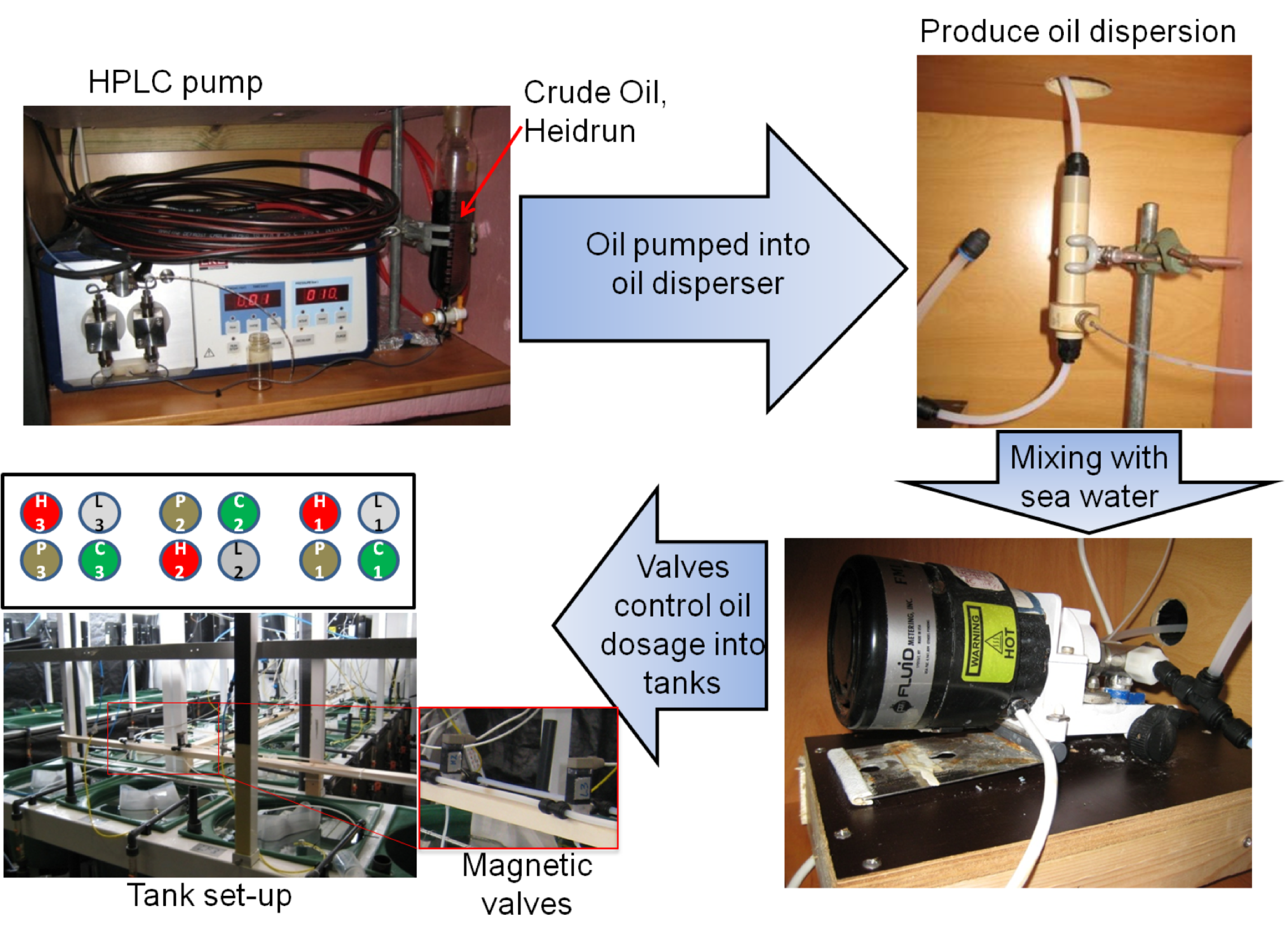

Supplement: S1 Fig — The oil was pumped into the dispersion system using a HPLC pump (Pharmacia, LKB2150). This system generates an oil dispersant with oil droplets in the low μm ranges. The exposure dose to the tanks was regulated by a parallel pipeline system with one line with clean sea water and one line with the dispersed oil—the 2 pipelines are connected by 3-way magnetic valve which switched between oil dispersant and clean water. (TIFF) [file pone.0124376.s001.tiff]

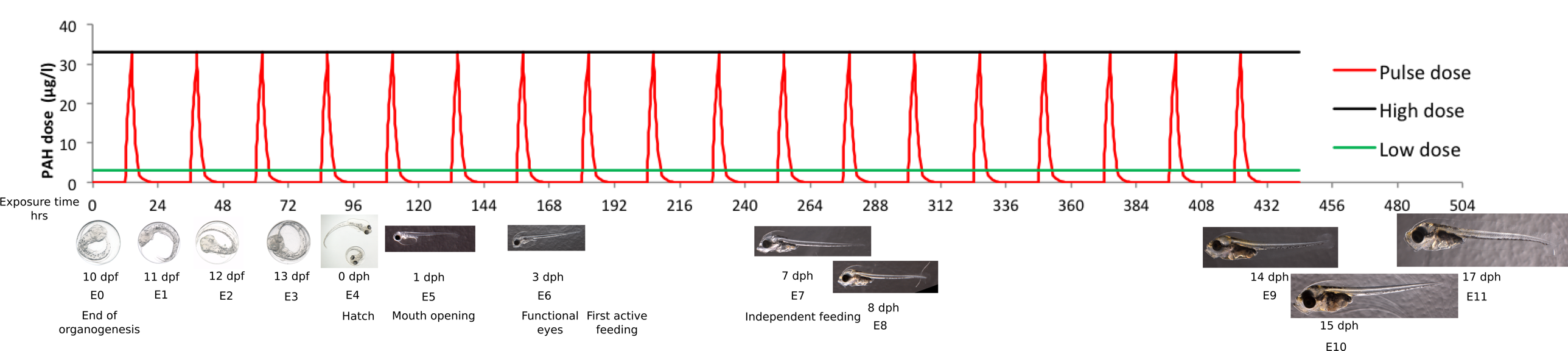

Supplement: S2 Fig — Oil exposure started at 10 dpf and ended at 14 dph after 18 days of exposure. The twelve sampling points are indicated by E0–E11. Low dose (green): nominal doses; 130 μg oil/L. High dose (black): nominal doses 1200 μg oil/L. Pulse dose (red): nominal doses 1200 μg oil/L for 2.4 hours in a 24 hour period. Concentration of oil in the pulse tank decreased to approx. 0 before next pulse. (TIFF) [file pone.0124376.s002.tiff]

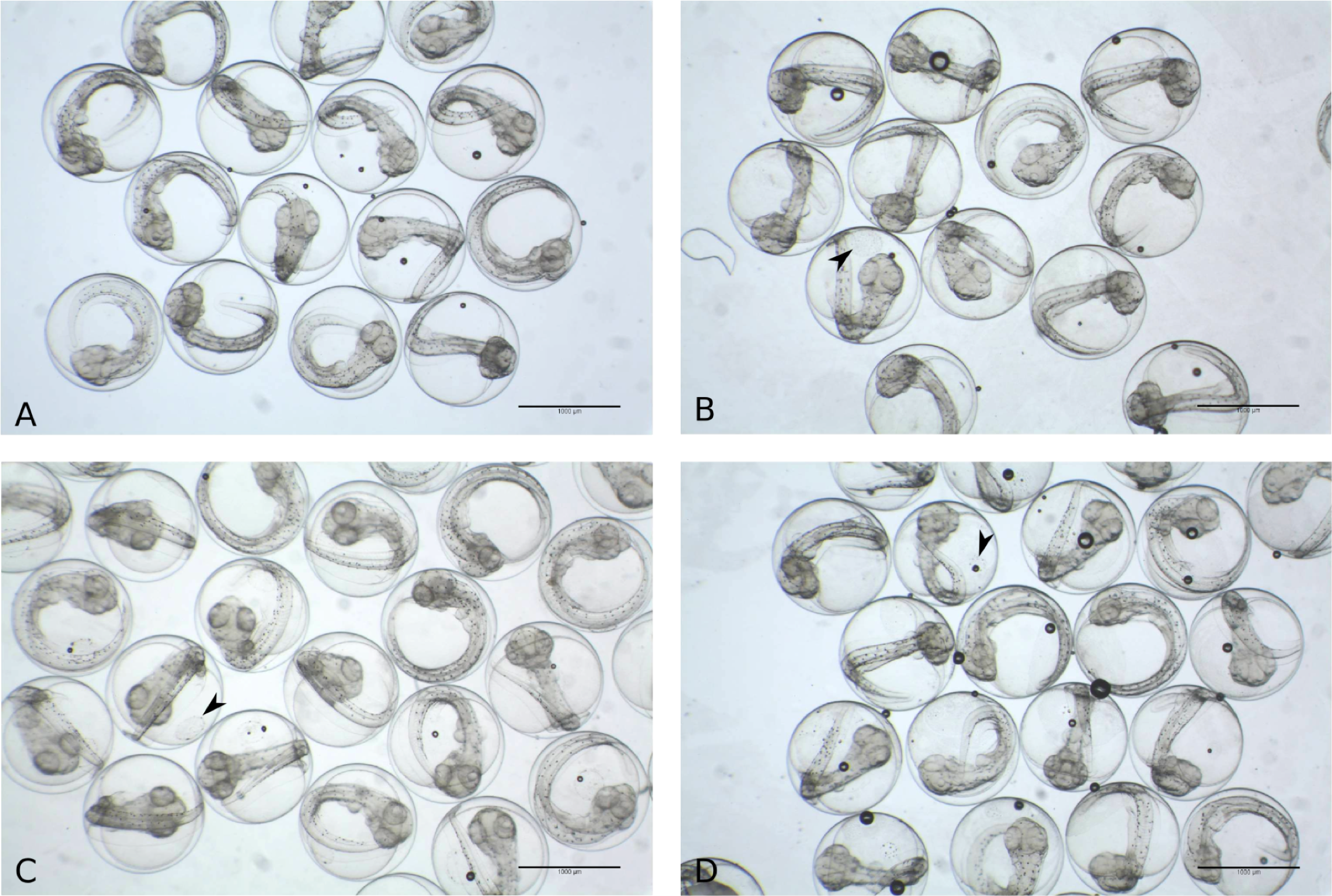

Supplement: S4 Fig — Micro-droplets of dispersed oil adhere to the chorion, and are observed in all exposure groups after 24 hours of exposure. Arrows indicate examples of micro-droplets adhered to the chorion of the Atlantic haddock embryo. (TIFF) [file pone.0124376.s004.tiff]

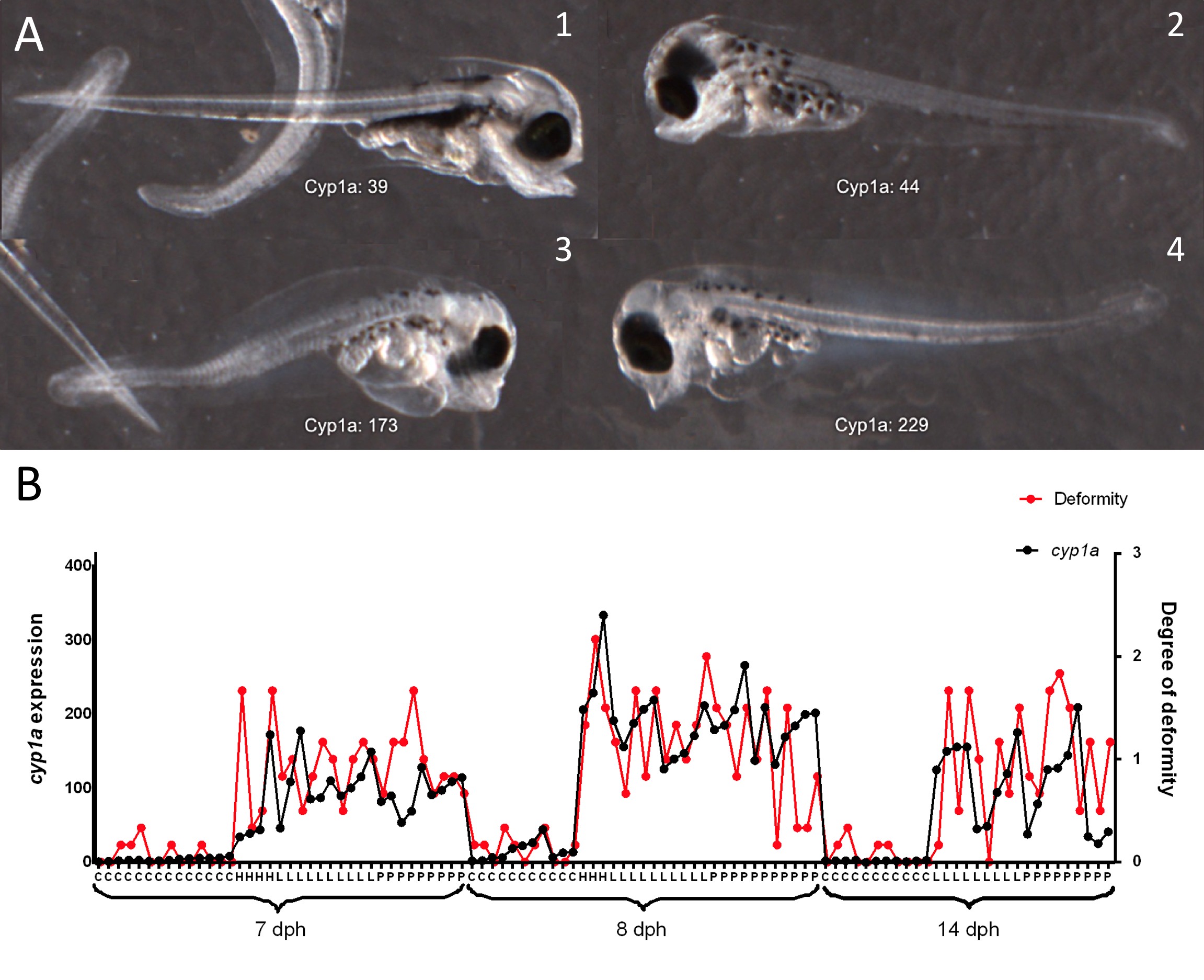

Supplement: S5 Fig — A: High dose larvae with fewer deformities tend to have lower expression level of cyp1a (1 and 2) compared to larvae with significant to severe deformities (3 and 4). The numbers indicate fold change in cyp1a expression compared to control. B: The graph shows the cyp1a expression (black curve) and the degree of deformity (red curve) for individuals with linked deformity-, cyp1a expression information. (TIFF) [file pone.0124376.s005.tiff]
