# Supplementary material for: Unexpected Interaction with Dispersed Crude Oil Droplets Drives Severe Toxicity in Atlantic Haddock Embryos
Source: PLoS One. 2015 Apr 29;10(4):e0124376. doi: 10.1371/journal.pone.0124376 (PMC4414579; doi:10.1371/journal.pone.0124376)
Supplement: S1 Table — (DOCX) [file pone.0124376.s007.docx]

| **Gene** | **Forward primer 5`-3`** | **Reverse primer 5`-3`** | **Probe 5`-3`** | **Quencher** |
| --- | --- | --- | --- | --- |
| *cyp1a* | CCTCCTTCCTGCCCTTCAC | TTGGGAATGAAGTAGCCATTGA | 6FAM-CCTCACTGCGCCACAAAAGACACATC | Tamra |
| *ahr2* | CAAACTCTATGCAGCCGAACAA | TCGCTGCTGCACTGCAA | 6FAM-TGGAGACGTGTCGGC | none |
| *gstp1* | GGTGCTGGTGAGCATGGAA | GAACACACAGGTGGCCTTCA | 6FAM-AATGGCAGAAGGGAG | none |
| ef1α | ATCGGCGGTATCGGAACAG | GCTTGAGGACACCGGTCTCA | 6FAM-ACCCGTGGGCCGTG | none |
