# Supplementary material for: Unexpected Interaction with Dispersed Crude Oil Droplets Drives Severe Toxicity in Atlantic Haddock Embryos
Source: PLoS One. 2015 Apr 29;10(4):e0124376. doi: 10.1371/journal.pone.0124376 (PMC4414579; doi:10.1371/journal.pone.0124376)
Supplement: S2 Table — The table shows the detailed numbers of the measurement that are combined in Fig 6 in the paper. All parameters have been graded 0–3, where 0 = no deformity, 1 = some deformity, 2 = significant deformity, 3 = severe deformity or as NA (not applicable) if position of larva made deformity grading difficult or impossible. * = no mouth opening yet. ** = too small to observe pericardial edema. The number shows the % of larvae that are scored from 1–3. (DOCX) [file pone.0124376.s008.docx]

| **Sampling point** | **Group** | **Craniofacial deformity (%)** | **(N_CD_)** | **Jaw deformity (%)** | **(N_JD_)** | **Yolk sac edema (%)** | **(N_Y_)** | **Spinal curvature (%)** | **(N_S_)** | **Pericardial edema (%)** | **(N_P_)** | **Lack of pigmentation (%)** | **(N_L_)** |
| --- | --- | --- | --- | --- | --- | --- | --- | --- | --- | --- | --- | --- | --- |
| 1 dph | C | 8 | (39) | * | 0 | 8 | (65) | 20 | (66) | ** | 0 | 8 | (62) |
| 1 dph | L | 42 | (43) | * | 0 | 52 | (83) | 30 | (70) | ** | 0 | 34 | (83) |
| 1 dph | P | 50 | (42) | * | 0 | 46 | (75) | 23 | (65) | ** | 0 | 48 | (71) |
| 1 dph | H | 41 | (17) | * | 0 | 88 | (89) | 17 | (56) | ** | 0 | 42 | (92) |
| 3 dph | C | 0 | (29) | 0 | (29) | 0 | (35) | 8 | (58) | 0 | (26) | 3 | (59) |
| 3 dph | L | 53 | (36) | 48 | (23) | 65 | (50) | 16 | (80) | 95 | (22) | 27 | (83) |
| 3 dph | P | 38 | (45) | 42 | (31) | 56 | (60) | 15 | (80) | 75 | (23) | 45 | (80) |
| 3 dph | H | 76 | (50) | 98 | (49) | 95 | (62) | 37 | (68) | 89 | (19) | 24 | (68) |
| 7 dph | C | 0 | (34) | 0 | (34) | 0 | (34) | 9 | (33) | na | (19) | 26 | (33) |
| 7 dph | L | 62 | (26) | 36 | (20) | 84 | (31) | 25 | (36) | 95 | (22) | 69 | (35) |
| 7 dph | P | 67 | (24) | 52 | (21) | 90 | (30) | 26 | (34) | 95 | (20) | 71 | (35) |
| 7 dph | H | 94 | (17) | 72 | (18) | 56 | (18) | 78 | (18) | 78 | (8) | 95 | (19) |
| 8 dph | C | 0 | (30) | 6 | (31) | 12 | (33) | 18 | (34) | 12 | (25) | 28 | (36) |
| 8 dph | L | 57 | (28) | 63 | (27) | 83 | (30) | 50 | (36) | 100 | (25) | 83 | (36) |
| 8 dph | P | 76 | (25) | 50 | (24) | 81 | (27) | 42 | (33) | 81 | (21) | 81 | (36) |
| 8 dph | H | 100 | (7) | 100 | (7) | 100 | (9) | 58 | (12) | 100 | (6) | 100 | (12) |
| 14 dph | C | 0 | (32) | 6 | (32) | 0 | (32) | 11 | (38) | 0 | (23) | 22 | (39) |
| 14 dph | L | 58 | (12) | 38 | (13) | 85 | (13) | 46 | (13) | 91 | (11) | 77 | (13) |
| 14 dph | P | 81 | (21) | 71 | (21) | 76 | (21) | 68 | (22) | 100 | (19) | 82 | (22) |
| 14 dph | H | - | - | - | - | - | - | - | - | - | - | - | - |
