# Supplementary material for: Unexpected Interaction with Dispersed Crude Oil Droplets Drives Severe Toxicity in Atlantic Haddock Embryos
Source: PLoS One. 2015 Apr 29;10(4):e0124376. doi: 10.1371/journal.pone.0124376 (PMC4414579; doi:10.1371/journal.pone.0124376)
Supplement: S1 Text — (DOC) [file pone.0124376.s009.doc]

**Analytical chemistry methods.**

Water samples from each tank (1 L) were taken before exposure start and after the end of exposure. Each sample was acidified with concentrated hydrochloric acid and added deuterated PAHs as internal standards (naphthalene-d8, biphenyl-d10, acenaphthylened8, anthracene-d10, pyrene-d10, perylene-d12 and indeno[123-cd]pyrene-d12 (200 ng in 100 µl hexane solution)) before being extracted with dichloromethane (DCM) and prepared for analysis of total hydrocarbons (THC) and PAHs. The samples were extracted by liquid-liquid extraction using 3x 30 mL DCM, before the combined extracts were dried over Na_2_So_4_. DCM extracts were reduced using a rotary evaporator and exchanged into isooctane to a final volume 0.5 mL. The THC was measured on a Agilent Technologies 6890N gas chromatograph with flame ionization detector (GC-FID) using a Varian Select™ Mineral Oil column (15 m x 0.32 mm, 0.10 µm). Instrumental parameters were; injector temperature 290ºC; detector temperature 275ºC; column temperature, 60ºC for 1 min, 60-290ºC at 20ºC/min, 24min at final temperature, carrier gas He at 1.4 mL/min. The quantification of THC was carried out using a 10 point external standard curve made from dilution of weathered Heidrun oil (4 ng/µl-2000 ng/µl) and the hump were integrated from C12-C35.

The PAHs were analyzed on a Micromass Autospec Ultima GC/MS in single ion monitoring mode. The GC/MS system was equipped with a HP-6890 GC, a 50m x 0,25mm, 0.25μm Varian Factor Four CC VF-5ms capillary column inserted directly into the ion source. Other conditions were: injector temperature 280ºC; transfer line 275ºC; column temperature, 60ºC for 1 min, 60-100ºC at 15ºC/min, 100-280ºC at 6ºC/min, 9min at final temperature, carrier gas He at 1.5 mL/min. Electron impact ionization at 70eV was used. Samples were injected by auto sampler, 1 μl splitless injection. The following PAH compounds were measured: C0, C1-, C2-, C3-, C4-naphthalenes (N, N1, N2, N3, N4), anthracene (ANT), acenaphthylene (AC), acenaphtene (AE), fluorene (F), C0-, C1-, C2-, C3- phenanthrene/anthracenes (P, P1, P2, P3), C0-, C1-, C2-, C3-dibenzothiophenes (D0, D1, D2, D3), fluoranthene (FL), pyrene (PY), benzo[a]anthracene (BAA), chrysene (C), 1-methylchrysene (C1), 6-ethylchrysene (C2), 6-propylcrysene (C3), Benzo[b]fluoranthene (BBF), benzo[k]fluoranthene (BKF), benzo[e]pyrene (BEP), benzo[a]pyrene (BAP), perylene (PER), benzo[ghi]perylene (BZP), indeno[1,2,3-cd]pyrene (IND) and dibenzo[a,h]anthracene (DBA). Parent PAH were analyzed in selected ion monitoring mode (SIM) by ion masses m/z 128, 152, 154, 178, 192, 202, 206, 228, 252, 276 and 278. Alkylated PAH were analysed by selective ion masses m/z 142, 156, 170, 184 (N1-N4), 192, 206, 220, (P1-P3), 198, 212, 226 (D1-D3), 242, 256, 270 (C1-C3) and integrated by baseline integration of the established chromatographic pattern and the response factors were assumed equal within each group of homologues. The deuterated PAHs were analysed by ion masses m/z 136, 164, 160, 188, 212, 264. 288.
